# High Iodine Deficiency among Pregnant Women in Periurban Ghana: A Hospital-Based Longitudinal Study

**DOI:** 10.1155/2018/9706805

**Published:** 2018-06-03

**Authors:** David Larbi Simpong, Yaw Asante Awuku, Kenneth Kwame Kye-Amoah, Martin Tangnaa Morna, Prince Adoba, Stephen Kofi Anin, Patrick Adu

**Affiliations:** ^1^Department of Medical Laboratory Science, University of Cape Coast, Cape Coast, Ghana; ^2^Department of Medicine, University of Cape Coast, Cape Coast, Ghana; ^3^Sefwi Wiawso Municipal Hospital in Ghana, Western Region, Ghana; ^4^Department of Surgery, University of Cape Coast, Cape Coast, Ghana; ^5^Trauma and Specialist Hospital, Winneba, Ghana; ^6^Department of Industrial and Health Science, Takoradi Technical University, Takoradi, Ghana

## Abstract

**Background:**

Iodine deficiency causes maternal hypothyroidism which can lead to growth, cognitive, and psychomotor deficit in neonates, infants, and children. This study examined the iodine status of pregnant women in a periurban setting in Ghana.

**Methods:**

This longitudinal study recruited 125 pregnant women by purposeful convenience sampling from the antenatal clinic of the Sefwi Wiawso municipal hospital in Ghana. Urinary iodine concentration (UIC) was estimated by the ammonium persulfate method at an estimated gestational age (EGA) of 11, 20, and 32 weeks. Demographic information, iodized salt usage, and other clinical information were collected using a questionnaire.

**Results:**

The prevalence of iodine deficiency among the pregnant women was 47.2% at EGA 11 and 60.8% at both EGA of 20 and 32, whereas only 0.8% of participants not using iodized salt had iodine sufficiency at EGA 32. 18.4%, 20%, and 24% of participants using iodized salt had iodine sufficiency at EGA 11, 20, and 32, respectively.

**Conclusion:**

A high prevalence of iodine deficiency was observed among our study cohort.

## 1. Introduction

Iodine is an essential nutrient used in the synthesis of thyroid hormones that are important for metabolism and the development of the nervous system [1]. Insufficient intake of iodine causes iodine deficiency disorders (IDD) including hypothyroidism [2]. Nearly, two billion (28%) of the world's population, of whom more than 321 million (39%) are Africans, are at risk of insufficient iodine intake [3]. Iodine deficiency is the leading and most preventable cause of mental retardation in the world, with a 10%–15% reduction in intelligent quotient [4].

Pregnant women require more iodine due to fetal iodine transfer, as well as the higher synthesis and subsequent transfer of maternal thyroid hormone to the fetus [5]. Iodine deficiency during pregnancy has been associated with maternal hypothyroxinemia that is related to growth, cognitive, and psychomotor deficit in neonates, infants, and children [6]. Severe iodine deficiency during pregnancy has been linked to the risk of spontaneous abortion, stillbirth, reduced intelligence, neurological cretinism, poor cognitive functions, and delayed psychomotor developments [1, 2, 7].

Though universal salt iodization (USI) has been recommended as the most cost-effective strategy to eliminate iodine deficiency disorders [8, 9], a surveillance program is necessary to evaluate the success of this initiative. Despite the high risk of iodine deficiency in sub-Saharan Africans, not many studies have focused on its prevalence and effects on pregnancy in Ghana. Most iodine-based research in Ghana employs cross-sectional study design and therefore presents only snap shots of the situation. This longitudinal study therefore sought to examine the iodine status of pregnant women at certain gestational age within the trimesters.

## 2. Methods

### 2.1. Study Design, Site, and Study Population

This prospective study was conducted from August 2015 to May 2016 at Sefwi Wiawso, a periurban setting in the Western Region of Ghana. The Sefwi Wiawso municipality has a total population of 139,200 with males and females constituting 50.1 percent and 49.9 percent of the population, respectively. The municipality has a total fertility rate of 3.7, which implies that on the average, a female aged 15–49 year will give birth to about 4 children by the end of her reproductive years [10].

### 2.2. Sample Size

**Figure 1:**
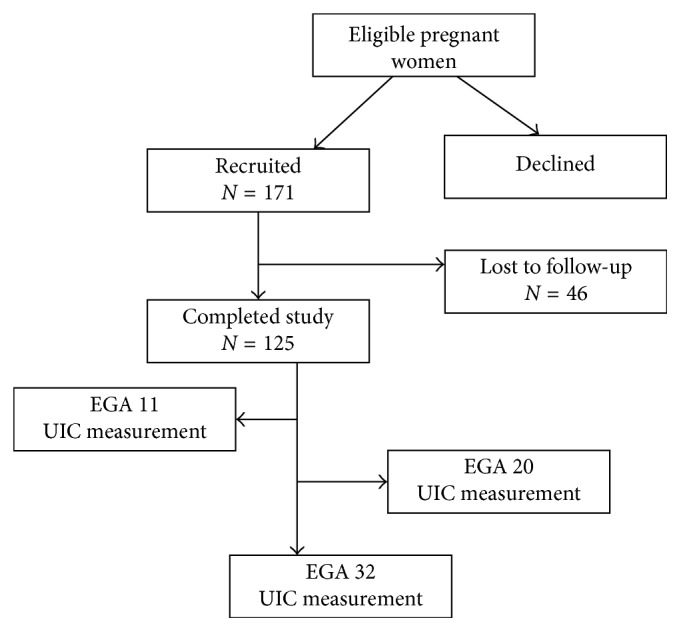
Participant recruitment. The final sample population comprised 125 pregnant women recruited in at EGA 11 (trimester 1) and followed-up through EGA 20 (trimester 2) to EGA 32 (trimester 3) by measuring UIC.

A total of 171 consecutive pregnant women who consented were initially recruited for this study; however, 46 were lost to follow-up (Figure 1). Overall, one hundred twenty-five (125) pregnant women attending the antenatal clinic of the Sefwi Wiawso municipal hospital completed the study. These pregnant women were recruited in the first trimester and followed-up through the second and third trimesters. Within this period, each participant's urine sample was measured for UIC, once in every trimester, at estimated gestational age (EGA) of 11, 20, and 32 weeks. All pregnant women with known chronic disorders or who smoked were excluded from the research.

### 2.3. Ethical Consideration

Ethical approval was sought from the University of Cape Coast Institutional Review Board (UCCIRB) and the authorities of the Sefwi Wiawso municipal hospital before commencement of the study. Informed written consent was also sought from the participants before taking their data and samples.

### 2.4. Collection and Determination of Urine Iodine Levels

Participants were provided with clean containers into which they provided on-the-spot urine for the measurement of urine-iodine concentration. Urine-iodine concentration was estimated by the ammonium persulfate method [11]. As a quality control measure, the interval between the time of addition of ceric ammonium sulfate and the reading the absorbance were all the same for all samples, standards, and blanks to rule out any systematic or random biases. Median urinary iodine concentration levels were defined as follows: excessive >500 *µ*g/l, above requirement 250–499 *µ*g/l, sufficient 150–249 *µ*g/l, and iodine insufficient (deficiency) <150 *µ*g/l [11].

### 2.5. Data Analyses

All data were analyzed using SPSS version 16 (IBM Corp.) and Minitab version 16 (Minitab Inc.). Data were analyzed using simple descriptive statistics such as frequency and percentages. Line graphs were also used to explore trends in the median UIC among age categories at EGA 11, 20, and 32 weeks.

## 3. Results

The measured median UIC among participants were categorized and compared to the standard median iodine concentration levels categorizations [12] as shown in Table 1. Excessive iodine levels decrease among participants as EGA increases. Eight percent pregnant women had excessive iodine level at EGA 11, *4%* at EGA 20 and *2.4%* at EGA 32. However, the percentage of pregnant women with sufficient iodine increased as EGA progresses (18.4% at EGA 11 to 25.6% at EGA 32). The percentage of iodine deficiency increased from 47.2% at EGA 11 to 60.8% at both EGA 11 and 32 among the participants (Table 1).

**Figure 2:**
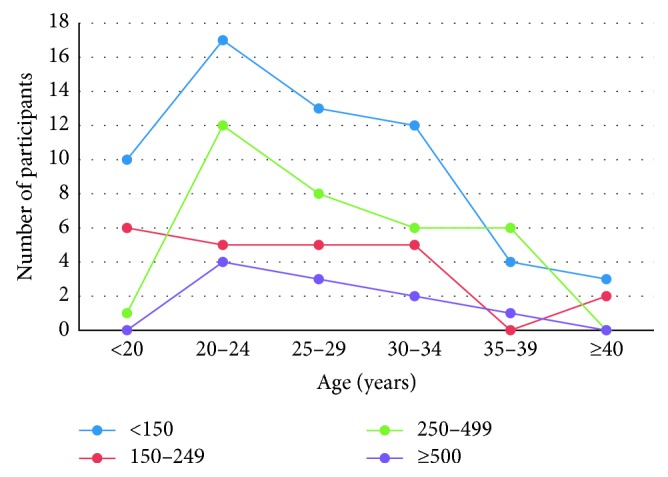
Participants' median UIC in relation with age at EGA 11.

Based on age, 17 participants were less than 20 years, 38 were between 20 and 24 years, 29 were between 25 and 29 years, 25 were between 30 and 34 years, 11 were between 35 and 39, and 5 participants were 40 years and above. The distribution of EGA 11 pregnant women's median urinary iodine concentration within a defined age group under a defined standard categorization of median urinary iodine concentration is shown in Figure 2.

**Figure 3:**
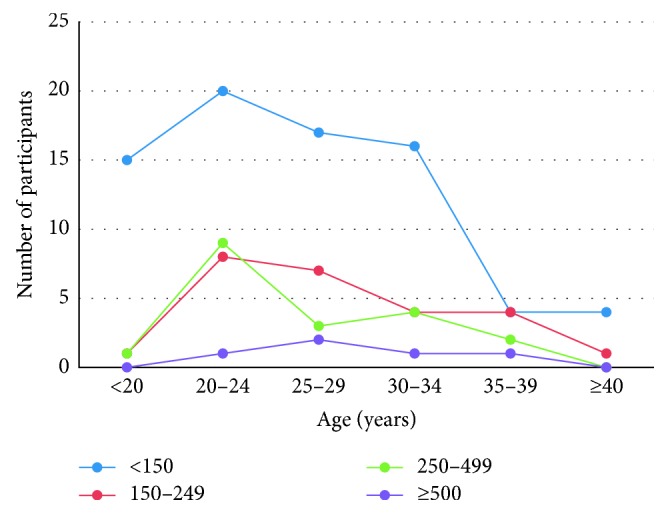
Participants' median UIC in relation with age at EGA 20.

The distribution of EGA 20 pregnant women's median urinary iodine concentration within a defined age group under a defined standard categorization of median urinary iodine concentration is shown in Figure 3. Iodine deficiency is observed to increase as the gestational age progressed among the same age group.

**Figure 4:**
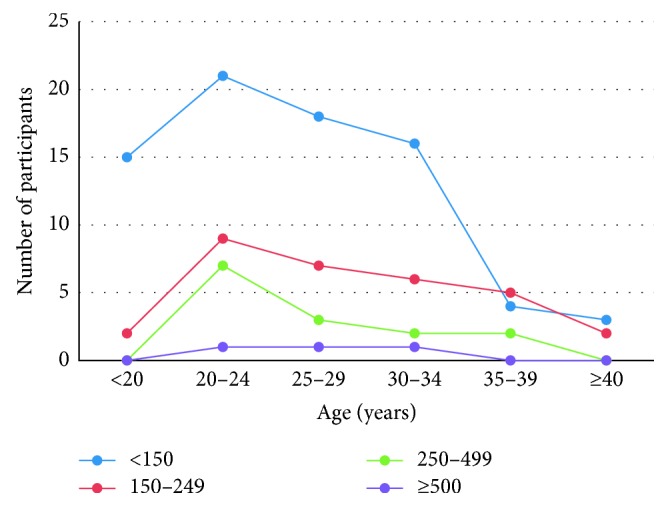
Participants' median UIC in relation with age at EGA 32.

The distribution of EGA 32 pregnant women's median urinary iodine concentration within a defined age group under a defined standard categorization of median urinary iodine concentration is shown in Figure 4.

Iodine deficiency was the highest among participants who did not use iodized salt (Table 2). Overall, none of the participants not using iodized salt had either iodine excess or above iodine requirement. Additionally, iodine deficiency among participants using iodized salt increased across the trimesters (1.6% at EGA 11 versus 15.2% EGA 20 versus 16.8% at EGA 32). Moreover, the proportion of participants having excess/above iodine levels decreased as the pregnancy progressed through the semesters, whereas only 1.6% of the participants who used iodized salt were iodine deficient at EGA 11, and 45.6% of participants not using iodized salts were iodine deficient.

## 4. Discussion

This longitudinal study sought to examine the iodine status of pregnant women in a periurban setting in Ghana. Excess or deficient iodine levels in an individual are characterized with varied pathology; hence, it is important to carefully interpret data to inform the best management approach. A previous publication on iodine status in the western region of Ghana reported that, of the salts sold in the market, only 58% were iodized, with an estimated iodine content of 20 ppm being far below the mandated requirement [13]. Regarding IDD, pregnant women are among the high-risk population and are therefore of interest to consider. Iodine level in an individual may vary from time to time; however, because pregnant women in Ghana generally attend antenatal clinic until delivery, it is easy to follow them for remeasurement of their UIC. Iodine deficiency was prevalent among 47.2% of the pregnant women who were in their EGA 11 and a prevalence of 60.8% at both EGA 20 and EGA 32, suggesting a higher iodine deficiency among our study cohort. Potential factors may include either the pregnant women consumed iodized salt with less iodine content and/or lack of consumption of appropriate amount of iodine in their daily meals. The former is more likely considering the fact that the salt sold in this part of Ghana contains less iodine content [13]. Taken together, we speculate that pregnant women and their babies in this part of Ghana are susceptible to iodine deficiency-related disorders in spite the educational campaigns embarked by the Ghana Ministry of Health, Ghana Health Service, Food and Drugs Authority of Ghana, Ghana Standard Authority, and World Health Organization on the use of iodized salt. Of interest in this study is the observed high iodine deficiency in the EGA 11. These findings are suggestive that most of the reproductive age women in our study population are iodine deficient during the preconception period and therefore call for the need to evaluate the effectiveness of these public health campaigns to address potential problem areas to meet set targets.

Gestation produces a physiological increase in the elimination of iodine in the urine because of a rise in glomerular filtration [14]. The findings reported herein are in support of the previous findings, and thus suggest that an alternative way of restoring iodine levels in pregnant women is vital. It is important to note that, even among the same age group, iodine deficiency increased as gestational age progressed. This may pose danger to both the mother and the fetus if possible management modalities are not employed.

As the entry point in our study was EGA 11, we missed the dynamics of iodine levels in the early part of gestation. It will be very interesting to screen prospective mothers for their iodine levels prior to becoming pregnant. This will help identify those who require iodine supplementation to avoid missing the crucial early stage of pregnancy where iodine levels ought to be sufficient.

Noncompliance with the use of iodized salt appears to have a significant influence on the iodine levels of the study participants. This is buttressed by the finding that only few of our study participants using iodized salt had iodine deficiency compared to those not using iodized salt. To prevent iodine deficiency, the American Thyroid Association recommends supplementation containing 150 *µ*g of iodine daily for women of childbearing age during the preconception phase as well as during pregnancy and lactation [15]. In addition, adequate iodine intake before conception (150 *µ*g per day) is important to ensure adequate maternal iodine stores to support the fetus. The preconception iodine status of the mother is likely to influence the degree of successful maturation of the fetal central nervous system and subsequent neurodevelopment of the child [16]. Following our previous work [17] and this present study, we propose the inclusion of iodine levels estimation in the routine laboratory screening for pregnant women in Ghana. This will help mirror the exact picture of iodine levels in these subjects and further help identify potential candidates who require iodine supplement during and after pregnancy. Also, continuous education through the media on the use of iodized salt should be intensified and, if possible, the presentation should be made in the local dialect so that all categories of the population would benefit from such campaigns.

The strength of this study lies in the fact that each pregnant woman was followed at certain defined gestational periods (11, 20, and 32 weeks), and therefore, likely to present the true picture of iodine levels in these subjects. Also, the quantitative method of iodine measurement was adopted, where chloric acid was used to digest the urine under mild condition and the catalytic role, the reduction of ceric ammonium sulfate determined [11]. This method is sensitive, less expensive, requires less labour, and can be applicable for a large-scale screening. Though the study has strength in being a longitudinal study to assess iodine levels among pregnant women in Ghana, it is also limited by the nondetermination of the impact of the iodine levels estimated herein on pregnancy outcomes.

## 5. Conclusion

A high prevalence of iodine deficiency was observed among the pregnant women visiting the periurban hospital in the western region of Ghana. It is important to reconsider why iodine deficiency increases as gestational age progressed among these populations and reevaluate the campaign on the use iodized salt within the region.

## Figures and Tables

**Table 1:** Iodine levels among pregnant women in the trimesters.

Criteria	Standard iodine concentration (*µ*g/Ɩ)	*N* = 125 (%)		
		EGA 11, *n* (%)	EGA 20, *n* (%)	EGA 32, *n* (%)
Excessive	**≥500**	10 (8.0)	5 (4.0)	3 (2.4)
Above requirement	**250–499**	33 (26.4)	19 (15.2)	14 (11.2)
Iodine sufficient	**150–249**	23 (18.4)	25 (20.0)	32 (25.6)
Iodine insufficient (deficient)	**<150**	59 (47.2)	76 (60.8)	76 (60.8)

EGA: estimated gestational age; *n*: number of participants.

**Table 2:** Iodine level in relation to iodized salt usage.

UIC	EGA 11		EGA 20		EGA 32	
(*µ*g/l)	Yes	No	Yes	No	Yes	No
≥500	10 (8.0)	0 (0.0)	5 (4.0)	0 (0.0)	3 (2.4)	0 (0.0)
250–499	33 (26.4)	0 (0.0)	19 (15.2)	0 (0.0)	14 (11.2)	0 (0.0)
150–249	23 (18.4)	0 (0.0)	25 (20.0)	0 (0.0)	30 (24.0)	1 (0.8)
<150	2 (1.6)	57 (45.6)	19 (15.2)	57 (45.6)	21 (16.8)	56 (44.8)

Yes: participants who consume iodized salt; no: participants who did not consume iodized salt.

## Data Availability

The data used to support the findings of this study are included within the article.
